# Analysis of the association of sugammadex with the length of hospital stay in patients undergoing abdominal surgery: a retrospective study

**DOI:** 10.1186/s12871-023-01979-4

**Published:** 2023-01-25

**Authors:** Jing Tan, Jianhua He, Lijun Wang, Jia Fang, Pengyi Li, Zhenghuan Song, Qingming Bian

**Affiliations:** 1 Department of Anesthesiology, Jiangsu Cancer Hospital & Jiangsu Institute of Cancer Research & The Affiliated Cancer Hospital of Nanjing Medical University, 42 Baizi Pavilion, Nanjing, Jiangsu Province China; 2 Department of Endocrine, Nanjing Hospital of Traditional Chinese Medicine, 157 Daming Road, Nanjing, Jiangsu Province China

**Keywords:** Sugammadex, Postoperative hospital stay, Reversal agents, Neuromuscular blockade

## Abstract

**Background:**

Sugammadex is a newer medication used for rapid and reliable reversal of neuromuscular blockade. This study evaluated whether sugammadex could reduce the length of postoperative hospital stay in patients undergoing abdominal surgery.

**Methods:**

This single center retrospective cohort study included patients who underwent major abdominal surgery between January 2015 and October 2019. Patients were randomized according to reversal with sugammadex or spontaneous recovery. The primary outcome was length of postoperative hospital stay. The secondary outcomes were length of post-anesthetic care unit (PACU) stay, postoperative ambulation time, time-to-first-defecation, and incidence of pulmonary complications. After 1:1 propensity score matching, univariate and multiple linear regression analyses estimated the differences in outcomes.

**Results:**

Of the 1614 patients, 517 received sugammadex and 645 spontaneously recovered. After adjusting for potential confounders, non-linear relationship was detected between administration of sugammadex and the length of postoperative hospital stay (β = 0.29 95% confidence interval {CI}: [− 1.13, − 0.54], *P* = 0.4912). However, it was associated with shorter PACU stay (β = − 20.30 95% CI: [− 24.48, − 17.11], *P* < 0.0001), shorter time to postoperative ambulation movement (β = − 0.43 95% CI: [− 0.62, − 0.23], *P* < 0.0001), and reduced time-to-first-defecation (β = − 2.25 95% CI: [− 0.45, − 0.05], *P* = 0.0129), when compared to the spontaneously recovered group. The incidence of pneumonia in the sugammadex group was significantly lower than that in the spontaneously recovered group (18.6% [44/237] vs. 39.2% [93/237] *P* < 0.05).

**Conclusions:**

Neuromuscular blockade reversal with sugammadex after abdominal surgery demonstrated an excellent recovery profile and was associated with decreased risk of pneumonia, although it did not affect the length of postoperative hospital stay.

**Supplementary Information:**

The online version contains supplementary material available at 10.1186/s12871-023-01979-4.

## Introduction

Neuromuscular blockade (NMB) is commonly used in anesthesia to facilitate endotracheal intubation and optimize surgical conditions, including during abdominal surgery [1]. Although short-acting muscle relaxants are widely used, residual NMB often occurs [2, 3]. Recent studies have reported that approximately 40% patients spontaneously recover [4, 5]. Incomplete reversal of NMB after emergence from anesthesia leads to increased incidence of postoperative complications [6], such as respiratory impairment [7–9]. Acetylcholinesterase inhibitors, already in clinical use, are ineffective in reversing deep muscle relaxation [10], and may be associated with high incidence of nicotinic and muscarinic side effects [8, 11].

Sugammadex is a cyclodextrin that selectively binds to free rocuronium (a neuromuscular blocker) molecules in plasma. Contrary to acetylcholinesterase inhibitors, sugammadex directly encapsulates rocuronium; hence, it facilitates rapid NMB reversal [11–13] without residual muscle relaxation [10, 14, 15]. Administration of sugammadex has been reported to expedite rapid recovery of smooth muscle functions, leading to improved postoperative bowel function, decreased recovery time, fewer postoperative pulmonary complications, and reduced costs [12, 16].

Enhanced recovery after surgery is garnering considerable attention. According to our observations, patients undergoing major abdominal surgery are at a high risk of perioperative respiratory complications due to their poor physical condition or multiple co-morbidities. Therefore, methods to ensure safe and effective rapid postoperative recovery are important considerations for surgeons and anesthesiologists.

Previous studies have reported that length of hospital stay is considered an important indicator for evaluating postoperative functional recovery [17] and is correlated with postoperative complications and use of resources [18]. However, despite the evidence to support the use of sugammadex, studies that determine its usefulness in reducing length of hospital stay after abdominal surgery are limited. Therefore, in this study, we investigated whether there was a difference in the length of postoperative hospital stay after administration of sugammadex when compared to spontaneous recovery after NMB for abdominal surgery and additionally, analyzed the differences in recovery by comparing gastrointestinal motility outcomes and postoperative pulmonary complication rates.

## Methods

### Ethical statements

This study was approved by the Ethics Committee of the Jiangsu Cancer Hospital on 30 March, 2020 (approval number: R-2020-039). Patient consent was waived because of the retrospective study design. The authors did not obtain information identifying individual participants during or after data collection.

In this single-center retrospective observational study, 1614 consecutive patients who underwent abdominal surgery in our institution between January 2015 and October 2019 (Time of patient recruitment) were enrolled. The baseline, intraoperative, postoperative, clinical, and follow-up data of each patient were collected and retrospectively reviewed in November 2020. Physical characteristics included age, body mass index (BMI) (kg/m^2^), and sex; preoperative comorbidities included American Society of Anesthesiologists (ASA) classification, hypertension, diabetes mellitus, and heart and cerebrovascular diseases; surgical characteristics included surgical duration and intraoperative rocuronium dose, type of surgery, and information regarding postoperative variables.

Patients with cancer who were scheduled for elective major abdominal surgery under general anesthesia with total intravenous anesthesia were included in the study. The exclusion criteria were as follows: (1) age < 18 years or > 70 years; (2) BMI ≥35 kg/m^2^; (3) ASA V or VI grade; (4) preoperative respiratory tract infection and other infectious diseases, chronic hepatorenal insufficiency, and severe perioperative complications; (5) organ transplantation surgery.

### Patient involvement

Only the medical records of patients were collected, identified, and reviewed. Therefore, patients were not involved in the design and conduct of the study, selection of outcome measures, or study enrollment.

### Sugammadex

Rocuronium, a neuromuscular blocking agent, was administered during major abdominal surgery. Following the operation, all patients were transferred to the PACU. Anesthetists could decide whether or not to use sugammadex according to their discretion. Time of administration of sugammadex depends on the anesthesia team’s clinical judgment [19]. Sugammadex was administered at 2 mg/kg, with a maximum of 200 mg per patient. The patients were extubated as soon as they awakened and were capable of following instructions. Tracheal extubation did not involve neuromuscular monitoring.

### Hospital and PACU stays

Length of stay was defined as the number of days in the postoperative hospital stay.

The length of post-anesthetic care unit (PACU) stay was the duration spent in the PACU. PACU discharge criteria were as follows: (1) awake and recovery of airway protective reflex; (2) hemodynamic stability; (3) recovery of spontaneous breathing, maintain airway patency, airway protective reflex recovery, respiration and oxygenation returned to preoperative basal levels (4) no obvious active bleeding, electrolyte and acid-base imbalances, and urine volume > 0.5 ml/kg/h.

Postoperative complications were confirmed through review of medical records and plain film radiographs or computed tomography images for the diagnosis of atelectasis and pneumonia.

### Statistical analysis

A propensity score matching (PSM) method was applied to minimize bias associated with confounding variables. Age (> 70 years), BMI score, ASA score (Classes 1, 2, and ≥ 3), preoperative comorbidities, duration of anesthesia, duration of surgery, intraoperative remifentanil dose, muscle relaxant dose, and type of surgery were matched as covariates. A 1:1 ratio matching was performed based on the propensity score with a standard caliper width of 0.2.

After confirming balance in the matched cohort with generalized linear models with a logarithmic link function, Wilcoxon signed-rank test was applied for propensity-matched patients; results of dichotomous variables were expressed as relative risk (RR) and 95% confidence intervals (95% CI). Mann-Whitney U test was used for continuous outcomes in all patients. Univariate and multiple linear regression analyses were applied after PSM to evaluate the difference between the groups of length of hospital stay, time-to-first-defecation, postoperative ambulation time, and length of PACU stay. *P*-value < 0.05 was considered statistically significant. (www.empowerstats.com, X&Y solutions, inc. Boston MA).

## Results

### Patients’ demographic data

This study was conducted between January 2015 and October 2019. Of the total of 1614 patients who underwent major abdominal surgery, 421 who were aged > 70 years, and 31 whose BMI were > 35 kg/m^2^ were excluded. Finally, 1162 patients were included in the analyses.

The demographic characteristics of the patients included in the study are presented in Supplementary Table 1. The covariate comparison groups are as follows: pre-PSM (sugammadex group: 517 and spontaneously recovered group: 645) and post-PSM (sugammadex group: 465 and spontaneously recovered group: 465). After 1:1 PSM, all the baseline characteristics were well-balanced, (absolute standardized difference ≤ 0.2) and the propensity score distribution was similar to PSM (Supplementary Table 1).

### Comparison of postoperative outcomes in the two groups

The results on the primary outcomes are presented in Table 1. Regarding postoperative recovery outcome, the length of postoperative hospital stay of patients in the sugammadex group was similar to that of the spontaneously recovered group (12.7 ± 6.4 days vs. 12.4 ± 7.3 days) (*P* = 0.07). This trend also did not change after PSM (12.75 ± 6.6 days vs. 12.40 ± 7.5 days) (*P* = 0.53).

**Table 1:** Primary postoperative outcomes before and after propensity score-matching

Variable	All patients before propensity score matching			All patients after propensity score matching		
	Recover spontaneously, *n* = 645	SGX, *n* = 517	*P*	Recover spontaneously, *n* = 465	SGX, *n* = 465	*P*
Postoperative hospital stay,days	12.70 (6.4)	12.36(7.3)	0.07	12.75 (6.6)	12.40(7.5)	0.53
The length of PACU stay, min	51.86(27.9)	32.82 (21.9)	*P* < 0.001*	52.56 (29.0)	32.77 (22.3)	< 0.0001*
Postoperative ambulation time, days	2.98(1.8)	2.57 (1.5)	*P* < 0.001*	2.99 (1.8)	2.52(1.3)	< 0.0001*
First feces’ time, days,	4.61(1.6)	4.38 (1.6)	*P* = 0.01*	4.58 (1.6)	4.34 (1.5)	0.0089*

The length of PACU stay were shorter in the sugammadex group than in the spontaneously recovered group and similar findings were observed in the propensity pair matched cohort (51.86 ± 27.9 vs 32.82 ± 21.9 min, 52.56 ± 29.0 vs 32.77 ± 22.3 min, respectively; *P* < 0.05 for all). The postoperative ambulation time in two groups has the similar finding (2.98 ± 1.8 vs 2.57 ± 1.5 days, 2.99 ± 1.8 vs 2.52 ± 1.3 days, respectively; *P* < 0.05 for all). The faster time-to-first-defecation following surgery also has the same trend in both groups (4.61 ± 1.6 vs 4.38 ± 1.6 days, 4.58 ± 1.6 vs 4.34 ± 1.5 days, respectively; *P* < 0.05 for all).

Regarding postoperative complications of matched cohorts in both spontaneously recovered and sugammadex groups (Table 2), the incidence of pneumonia was less in the sugammadex group than in the spontaneously recovered group before and after PSM (2.3 vs. 4.0%, *P* < 0.05 and 1.9 vs. 4.3%, *P* < 0.05, respectively). However, no differences were observed in other postoperative complications between the 2 groups.

**Table 2:** Postoperative outcomes before and after propensity score-matching

	All patients			All patients after propensity score matching		
Variable	Recover spontaneously, *n* = 645	SGX, *n* = 517	RR (95%CI)	Recover spontaneously, *n* = 465	SGX, *n* = 465	RR (95%CI)
Pneumonia (Yes)	645(4.0)	517 (2.3)	0.58	465(4.3)	465(1.9)	0.45
			(0.29,1.13) *			(0.21, 0.98) *
Atelectasis (Yes)	645(5.3)	517(4.5)	0.84	465(5.4)	465(4.3)	0.76
			(0.50,1.41))			(0.42, 1.36)

Data are N (%), The Fisher’s exact test for dichotomous outcomes in all patients, *OR* Odds ratio, CI Confidence interval, *SGX* Sugammadex

### Univariate analysis

The results of univariate analysis are presented in Supplementary Table 2. These suggested that age was related to longer PACU stay and longer time-to-first-defecation, whereas surgery duration, anesthesia duration, total remifentanil dose, and muscle relaxant dose were related to longer hospital stay. COPD and diabetes were positively related to an increase in postoperative length of hospital stay. Laparoscopic surgery was associated with reduced PACU stay and shorter length of postoperative hospital stay. These results suggested that anesthesia and surgery durations were more likely to influence length of postoperative hospital stay than administration of sugammadex alone. Intestinal resection had a shorter PACU stay and shorter time-to-first-defecation than gastric surgery. The length of postoperative hospital and PACU stays in the hepatic and pancreas surgery groups were significantly longer than in the gastric surgery group. However, the time-to-first-defecation for patients who underwent hepatic surgery was shorter than that for patients who underwent gastric surgery.

### Results of relationship between sugammadex with postoperative outcomes

Subsequently, we performed mixed-effects linear regression analysis for factors related to postoperative outcomes as presented in Supplementary Table 2. Three models were tested: the first was unadjusted for confounding factors; the second was adjusted for sex, age, BMI,and preoperative comorbidities; and the third was adjusted for surgery duration (minutes), muscle relaxant dose (mg), laparoscopic surgery, type of surgery, and total remifentanil dose. Each model confirmed that sugammadex was associated with shorter time to first flatus and first bowel movement, and shorter length of PACU stay. However, none of the 3 tested models identified an association of sugammadex with the length of postoperative hospital stay. (Table 3).

**Table 3:** Effect of sugammadex on postoperative recovery, based on a propensity score-matched cohorts

Outcome	Crude		Adjust I		Adjust II	
	β (95% CI)	*P* value	β (95% CI)	*P* value	β (95% CI)	*P* value
First feces’ time (days)	−0.24 (− 0.44, − 0.03)	0.0217	− 0.24 (− 0.44, − 0.04)	0.0188	−0.25 (− 0.45, − 0.05)	0.0129
Postoperative ambulation time	−0.46 (− 0.67, − 0.26)	< 0.0001	−0.48 (− 0.69, − 0.27)	< 0.0001	−0.43 (− 0.62, − 0.23)	< 0.0001
Postoperative hospital stay (days)	−0.35 (−1.26, 0.55)	0.4439	− 0.41 (− 1.32, 0.49)	0.3701	−0.29 (− 1.13, 0.54)	0.4912
The length of PACU stay (min)	−19.78 (−23.11, − 16.46)	< 0.0001	−19.96 (− 23.24, − 16.67)	< 0.0001	−20.30 (− 23.48, −17.11)	< 0.000

Non-adjusted model adjust for: None

Adjust I adjust for: sex; age; ASA; BMI; hypertension; diabetes; cerebral infarction; smoking history; drinking; heart disease

Adjust II adjust for: operation duration (minutes); dosage of muscle relaxant (mg); laparoscopic surgery; type of surgery; total remifentanil

## Discussion

Our study demonstrated that intraoperative administration of sugammadex significantly reduced the time to passage of flatus or feces and dramatically reduced PACU length of stay. However, there was no impact on the length of postoperative hospital stay.

The results of our study suggested that sugammadex has a significant effect on the recovery of gastrointestinal motility concurrent with previous studies [12, 20]. Conversely, some researchers have reported that sugammadex had limited influences on postoperative bowel movement. Sen et al. [12] reported that sugammadex does not significantly affect gastric emptying after abdominal surgery. Sustic, et al. [21] investigated the influence of sugammadex on gastric emptying time, measured by paracetamol absorption following laparoscopic surgery, and reported that sugammadex did not have a rapid and strong effect on gastric emptying. This is inconsistent with the findings of previous studies and may be due to several reasons as follows: differences in surgery [22] and anesthesia agents [23], consequence of intrinsic factors (e.g., the bidirectional communication between the central and enteric nervous systems and hormones [24, 25]); or pathologic conditions [24](e.g., when it is time to eat). In our study, the sugammadex group significantly reduced the time to first defecation and flatus. The results were further supported by An J et al. [26] who, in a prospective study, demonstrated quicker recovery of gastrointestinal motility in patients who received sugammadex following laparoscopic cholecystectomy surgery. Another retrospective cohort analysis by Hunt et al. [27] also validated these results. The results of his study demonstrated a faster recovery of bowel function and gastrointestinal motility after colorectal surgery with sugammadex compared with neostigmine/glycopyrrolate.

Nevertheless, in our study, people in sugammadex group received less opioids as compared to the spontaneously recovered group, Although remifentanil is a ultrashort effect opioid, considering the adverse gastrointestinal effects of opioid use [28]. This may partially influence our results.

We did not use the conventional drugs such as neostigmine as the control treatment. Neostigmine can promote intestinal peristalsis by the inhibition of acetylcholinesterase [29–31], .therefore, the effect of sugammadex on gut motility may have been more pronounced in comparison to our control population. Additionally, we used propensity score matching to mitigate potential sources of bias, and sugammadex was the major variable in our multivariable linear regression. Therefore, our results may suggest the positive impact of sugammadex on the passage of first flatus or feces.

Brueckmann et al. [32] reported that postoperative administration of sugammadex is effective for reversing residual NMB during recovery in PACU after abdominal surgery. Similarly, Carron et al. [33] conducted a systematic review and meta-analysis comparing postoperative discharge between patients administered sugammadex and neostigmine after general anesthesia and reported a reduction in time to discharge from PACU to the surgical ward for patients who underwent reversal with sugammadex. These results may be attributed to faster recovery from profound NMB after administration of sugammadex. Sugammadex can eliminate residual curarization and save costs associated with residual NMB management [34]. A muscle-spindle theory hypothesized that sugammadex has a stronger wake-promoting effect on arousal centers in the brain than neostigmine [35, 36]. A previous study reported that intraoperative administration of rocuronium reduces postoperative pain, the need for rescue analgesia, and length of PACU stay [37].

Although our results indicated that administration of sugammadex effected the early recovery of bowel function and contributed to the efficiency of PACU turn over, there was no difference in length of postoperative hospital stay between the sugammadex and spontaneous recovery groups in our study. This conclusion is currently debatable because it is not concurrent with the reported results of other studies. A recent retrospective study with a large sample size reported a trend toward a shorter hospital stay and lower rate of 30-day readmissions after administration of sugammadex for NMB reversal in patients undergoing major abdominal surgery [11]. Similarly, Watts et al. reported that sugammadex significantly reduced the length of hospital stay [5, 38]. Conversely, a retrospective study by Chae et al. [38] revealed that the total length of hospital stay and readmission rate of patients among 2 groups who were administered sugammadex and acetylcholinesterase inhibitors were not significantly different over 30 postoperative days. However, patients in the sugammadex group had a lower incidence of delayed discharge. The possible reasons for these inconsistent findings may be interactions between surgical and anesthesiologic management that can lead to clinically relevant consequences, postoperatively, and multiple factors that affect length of stay. Murphy et al. [39] reported that robot-assisted laparoscopic surgery resulted in a shorter hospital stay than laparoscopy or open surgery. Yeh et al. reported that diabetes increased length of hospital stay and 30-day postoperative mortality [40]. An earlier study suggests that age, surgery type, and laparoscopic or open surgery are predictive factors in increased postoperative hospitalization duration [41].

Consistent with the aforementioned results, our analysis revealed that pancreatic surgery and hepatobiliary resection contributed to increased length of hospital and PACU stays, though these durations were significantly shorter in laparoscopy than in open surgery. However, we did not conduct an accurate evaluation for different surgical sites and specific surgical approaches; hence, the differences in the results can be attributed to this reason. Factors such as social norms, reimbursement models, and the availability of reliable rehabilitation systems remain important parameters for length of stay. Thus, postoperative hospital stays partially reflect postoperative recovery [42], indicating that the result might be due to several of the aforementioned factors that affect length of postoperative hospital stay. Hence, additional research is needed to analyze the associations between sugammadex and length of postoperative hospital stay.

Early postoperative neuromuscular recovery with inadequate NMB is a frequent complication of anesthesia. This period was associated with either postoperative complications or intensive care unit stay following delayed recovery from anesthesia [39]. Postoperative pulmonary complications are more common after abdominal procedures [43].

Postoperative pulmonary complication rate and average time from operation to discharge were reported to be significantly reduced in patients administered sugammadex than those administered pyridistigmine [43, 44]. Ren M et al. compared the cost-effectiveness of using sugammadex versus neostigmine in laparoscopic surgery and reported that sugammadex decreased the incidence of postoperative respiratory complications and related costs [45]. Tsukada S et al. reported that administration of sugammadex in patients with myasthenia gravis after thymectomy was not associated with increased risk of respiratory complications [46]. Kirmeier E et al. report the results of a prospective European cohort study of 22,803 surgical in patients who received general anaesthesia for non-cardiac surgery, showed that the use of reversal agents could not decrease this risk [19].

Our study presents newer data that sugammadex minimizes residual NMB and results in a decreased incidence of pneumonia, but it could not reduce the incidence of other postoperative pulmonary complications. The conclusions from various studies are not uniform, because there are many mixed influencing factors such as type of surgical procedure, the patient’s own conditions, consumption of perioperative opioids, difference of surgical and anaesthetic techniques, which can all influence the incidence of postoperative pulmonary complications. Further studies are required to verify the beneficial effect of sugammadex on clinical respiratory outcomes.

Our study has some limitations. First, since this single-center study was conducted with a relatively small sample size, it is not fully representative of the groups as a whole. Therefore, future multicenter prospective studies with a large sample size are necessary. Second, only patients who had undergone abdominal surgery were included in the study and opioid may also has the potential for a certain impact on intestinal motility. Third, the long-term effects of surgery on postoperative outcomes were not observed. Therefore, future retrospective studies are needed to validate these results.

## Conclusions

The results of this study revealed that administration of sugammadex did not shorten hospital stay after abdominal surgery. Sugammadex was useful in decreasing recovery time in the PACU without an increase in pulmonary complications.

## Supplementary Information

**Additional file 1: Supplementary Table 1.** Characteristics of patients who underwent abdominal surgery before and after propensity score-matching. Presented as n (%) or mean (SD). **Supplementary Table 2.** Univariate relationship between patient characteristics and outcome, based on the propensity score-matched cohort.

## Data Availability

All data generated or analyzed during this study are included in this published article and its supplementary information file.

## Abbreviations

NMB: Neuromuscular blockade
ASA: American Society of Anesthesiologists
BMI: Body mass index
PACU: Post-anesthetic care unit
PSM: Propensity score matching
RR: Relative risk
CI: Confidence interval
