# Supplementary material for: Analysis of the association of sugammadex with the length of hospital stay in patients undergoing abdominal surgery: a retrospective study
Source: BMC Anesthesiol. 2023 Jan 25;23:32. doi: 10.1186/s12871-023-01979-4 (PMC9875499; doi:10.1186/s12871-023-01979-4)
Supplement: Supplementary file 1 — Additional file 1: Supplementary Table 1. Characteristics of patients who underwent abdominal surgery before and after propensity score-matching. Presented as n (%) or mean (SD). Supplementary Table 2. Univariate relationship between patient characteristics and outcome, based on the propensity score-matched cohort. [file 12871_2023_1979_MOESM1_ESM.pdf]

Supplementary Table 1 Characteristics of patients who underwent abdominal surgery before and after propensity score-matching. Presented as n (%) or mean (SD)

Variables	Before propensity score matching n = 1162			After propensity score matching n = 970		
	Recover spontaneously n = 645	SGX, n = 517	ASD	Recover spontaneously n = 465	SGX, n = 465	ASD
Age, year, mean (SD)	58.2 (8.8)	58.2 (8.9)	0.00	58.1(9.0）	58.0（8.8）	0.01
Sex, male, n (%)	418 (64.8)	339 (65.6)		303(65.2)	306 (65.8)	0.014
BMI, kg/m2, mean (SD)	23.9 (3.0)	23.8 (2.9)	0.04	23.8（3.1）	23.8（2.9）	0.00
ASA physical status, n (%)			0.27			0.05
Ⅰ	8 (1.2)	4 (0.8)		8 (1.7)	3 (0.6)	

Ⅱ	551 (85.4)	391 (75.6)		385(82.8)	363(78.1)	
Ⅲ	82 (12.7)	116 (22.4)		68(14.6)	96(20.6)	
Ⅳ	4 (0.6)	6 (1.2)		4(0.9)	3 (0.6)	
Preoperative comorbidities						
Hypertension, n (%)	212 (32.9)	164 (31.7)	0.02	146 (31.4)	141 (30.3)	0.02
Diabetes mellitus, n (%)	64 (9.9)	67 (13.0)	0.10	57 (12.3)	56 (12)	0.01
Heart disease, n (%)	24 (3.7)	59 (11.4) *	0.29	23 (4.9)	25 (5.4)	0.02
History of cerebral infarction, n (%)	22 (3.4)	20 (3.9)	0.02	18 (3.9)	16 (3.4)	0.02
Smoking status, n (%)	37 (5.7)	43 (8.3)	0.10	30(6.5)	35(7.5)	0.04
Drinking status, n (%)	26 (4.0)	31 (6.0)	0.09	22(4.7)	25(5.4)	0.03
COPD, n (%)	3 (0.5)	9 (1.7)	0.12	3 (0.6)	3 (0.6)	0.00
Chemotherapy history, n (%)	78 (12.1)	67 (13.0)	0.03	54(11.6)	60(12.9)	0.04

Operative characteristics, mean (SD)						
Duration of anesthesia, min	219.7 (81.1)	225.9 (79.1)	0.08	221.40 (82.90)	225.63 (78.58)	0.05
Muscle relaxant dosage, mg	152.7（59.6）	148.3（52.6）	0.02	151.1（51.3）	147.3（48.5）	0.08
Intraoperative remifentanil dose,mg	104.2（39.2）	89.7(35.4)	0.05	88.6（34.9）	89.4（35.0）	0.03
Duration of surgery, min	185.3 (75.8)	186.9 (74.2)	0.02	188.0 (78.2)	186.2 (74.38)	0.02
Type of surgery			0.14			0.17
Gastric Resection n (%)	235 (36.4)	201 (38.9)		159 (32.6)	188 (38.6)	
intestines resection n (%)	248 (38.5)	173 (33.5)		195 (40)	162 (33.3)	
Hepatobiliary resection n (%)	52 (8.1)	47 (9.1)		41 (8.4)	45 (9.2)	
Urinary operation n (%)	54 (8.4)	40 (7.7)		45 (9.2)	39 (8)	
pancreas operation n (%)	36 (5.6)	30 (5.8)		31 (6.4)	29 (6)	

others n (%)	20 (3.1)	26 (5.0)		16 (3.3)	24 (4.9)	
Laparoscopic surgery			0.10			0.01
0	532 (82.5)	405 (78.3)		388 (79.7)	387 (79.5)	
1	113 (17.5)	112 (21.7)		99 (20.3)	100 (20.5)	

ASA American Society of Anesthesiologists; BMI Body mass index; SGX sugammadex; ASD absolute value of standardized mean difference, Presented as n (%) or mean (standard deviation). For continuous variables: Mean ± SD, For categorical variables: n (%). Fisher precision for categorical variables, t-test for continuous variables; *: $P<0.05$.

Supplementary Table 2 Univariate relationship between patient characteristics and outcome, based on the propensity score-matched cohort

Covariate	Postoperative hospital stay (days)		The length of PACU stay (min)		First feces' time (day)	
	β (95% CI)	P value	β (95% CI)	P value	β (95% CI)	P value
Sex (female vs. male)	0.49 (-0.46, 1.43)	0.317	1.99 (-1.75, 5.73)	0.298	-0.11 (-0.33, 0.10)	0.298
Age, years, mean (SD)	0.05 (-0.00, 0.10)	0.054	0.53 (0.34, 0.73)	<0.0001*	0.02 (0.01, 0.03)	<0.0001*
BMI, kg/m^2, mean (SD)	-0.02 (-0.17, 0.13)	0.788	-0.22 (-0.81, 0.38)	0.473	0.01 (-0.03, 0.04)	0.657
ASA physical status						
II	reference		reference		reference	

I	-1.44 (-5.61, 2.73)	0.499	-9.16 (-25.61, 7.30)	0.276	0.01 (-0.93, 0.95)	0.984
III	1.21 (0.03, 2.40)	0.045	3.62 (-1.05, 8.29)	0.129	0.10 (-0.16, 0.37)	0.446
IV	4.37 (-0.84, 9.58)	0.101	9.36 (-11.21,29.94)	0.373	-0.45 (-1.62, 0.73)	0.457
Hypertension (yes vs. no)	0.25 (-0.72, 1.23)	0.610	4.99 (1.15, 8.83)	0.011*	0.07 (-0.15, 0.29)	0.530
Diabetes mellitus (yes vs. no)	1.45 (0.07, 2.83)	0.040*	2.23 (-3.21, 7.68)	0.422	0.03 (-0.28, 0.34)	0.849
Heart disease (yes vs. no)	1.15 (-0.89, 3.19)	0.270	3.03 (-5.01, 11.07)	0.460	0.24 (-0.22, 0.70)	0.304

History of cerebral (yes vs. no)	-1.42 (-3.83, 0.98)	0.246	7.64 (-1.82, 17.11)	0.114	-0.20 (-0.74, 0.34)	0.460
Smoking status (yes vs. no)	-0.60 (-2.37, 1.17)	0.504	-2.22 (-9.20, 4.75)	0.533	0.30 (-0.10, 0.70)	0.139
Drinking status (yes vs. no)	0.24 (-1.82, 2.31)	0.816	-1.96 (-10.08, 6.16)	0.637	0.10 (-0.36, 0.56)	0.678
COPD (yes vs. no)	6.80 (1.18, 12.42)	0.018*	-7.55 (-29.76,14.66)	0.506	0.71 (-0.55, 1.97)	0.270
Heart disease (yes vs. no)	1.15 (-0.89, 3.19)	0.270	3.03 (-5.01, 11.07)	0.460	-0.09 (-0.56, 0.37)	0.697

Duration of anesthesia, min, mean (SD)	0.02 (0.01, 0.02)	<0.0001*	-0.05 (-0.07, -0.03)	<0.0001*	0.00 (0.00, 0.00)	0.001*
Duration of surgery, min, mean (SD)	0.02 (0.02, 0.03)	<0.0001*	-0.04 (-0.06, -0.01)	0.003*	0.00 (0.00, 0.00)	<0.0001*
Dosage of muscle relaxant (mg)	0.03 (0.02, 0.03)	<0.0001*	-0.04 (-0.08, 0.00)	0.0263*	0.00 (0.00, 0.01)	0.0002*
Total remifentanil	0.04 (0.02, 0.05)	<0.0001*	-0.07 (-0.12, -0.01)	0.011*	0.00 (0.00, 0.01)	0.009*

Laparoscopic surgery (yesvs.no)	-1.46 (-2.58, -0.34)	0.0110*	0.42 (-4.01, 4.85)	0.853	-0.44 (-0.69, -0.19)	0.0006*
Type of surgery						
Gastric resection	reference		reference		reference	
intestines resection	-0.24 (-1.22, 0.74)	0.6343	-5.99 (-10.00, -1.98)	0.004	-0.53 (-0.76, -0.29)	<0.0001
Hepatobiliary resection	1.81 (0.27, 3.35)	0.0212*	16.88 (10.61, 23.16)	<0.0001*	-0.49 (-0.85, -0.12)	0.009*
Urinary operation	0.01 (-1.67, 1.69)	0.9908	12.38 (5.52, 19.24)	0.004	0.00 (0.00, Inf)	0.990

pancreas	10.80	<0.0001*	10.48	0.007*	0.10	0.662
operation	(8.94, 12.66)		(2.89, 18.08)		(-0.34, 0.54)	
Others	0.87	0.4394	-3.70	0.417	-0.22	0.400
	(-1.33, 3.06)		(-12.64, 5.23)		(-0.74, 0.30)	
